# Noninvasive hemodynamic indices of vascular aging: an in silico assessment

**DOI:** 10.1152/ajpheart.00454.2023

**Published:** 2023-09-22

**Authors:** Jingyuan Hong, Manasi Nandi, Peter H. Charlton, Jordi Alastruey

**Affiliations:** ^1^Division of Imaging Sciences and Biomedical Engineering, King’s College London, St. Thomas’ Hospital, London, United Kingdom; ^2^School of Cancer and Pharmaceutical Science, King’s College London, London, United Kingdom; ^3^Department of Public Health and Primary Care, University of Cambridge, Cambridge, United Kingdom

**Keywords:** arterial stiffness, database of virtual subjects, pulse wave, vascular aging, Young’s modulus

## Abstract

Vascular aging (VA) involves structural and functional changes in blood vessels that contribute to cardiovascular disease. Several noninvasive pulse wave (PW) indices have been proposed to assess the arterial stiffness component of VA in the clinic and daily life. This study investigated 19 of these indices, identified in recent review articles on VA, by using a database comprising 3,837 virtual healthy subjects aged 25–75 yr, each with unique PW signals simulated under various levels of artificial noise to mimic real measurement errors. For each subject, VA indices were calculated from filtered PW signals and compared with the precise theoretical value of aortic Young’s modulus (*E*_Ao_). In silico PW indices showed age-related changes that align with in vivo population studies. The cardio-ankle vascular index (CAVI) and all pulse wave velocity (PWV) indices showed strong linear correlations with *E*_Ao_ (Pearson’s *r*_p_ > 0.95). Carotid distensibility showed a strong negative nonlinear correlation (Spearman’s *r*_s_ < −0.99). CAVI and distensibility exhibited greater resilience to noise compared with PWV indices. Blood pressure-related indices and photoplethysmography (PPG)-based indices showed weaker correlations with *E*_Ao_ (*r*_p_ and *r*_s_ < 0.89, |*r*_p_| and |*r*_s_| < 0.84, respectively). Overall, blood pressure-related indices were confounded by more cardiovascular properties (heart rate, stroke volume, duration of systole, large artery diameter, and/or peripheral vascular resistance) compared with other studied indices, and PPG-based indices were most affected by noise. In conclusion, carotid-femoral PWV, CAVI and carotid distensibility emerged as the superior clinical VA indicators, with a strong *E*_Ao_ correlation and noise resilience. PPG-based indices showed potential for daily VA monitoring under minimized noise disturbances.

**NEW & NOTEWORTHY** For the first time, 19 noninvasive pulse wave indices for assessing vascular aging were examined together in a single database of nearly 4,000 subjects aged 25–75 yr. The dataset contained precise values of the aortic Young’s modulus and other hemodynamic measures for each subject, which enabled us to test each index’s ability to measure changes in aortic stiffness while accounting for confounding factors and measurement errors. The study provides freely available tools for analyzing these and additional indices.

## INTRODUCTION

Vascular aging (VA) describes the change in vascular structure and function over time. It is an initially asymptomatic process that can eventually lead to deterioration of cardiovascular function and end-organ damage. Quantification of vascular aging can capture the early features of vascular degeneration before they become symptomatic in later life (1), and therefore, objective measures of vascular aging may help identify individuals at elevated risk of cardiovascular disease, to support earlier interventions.

The concept of vascular aging is gaining interest clinically. Several indices of vascular aging have been proposed in clinical studies to assess atherosclerosis (narrowing of the arterial lumen) and arteriosclerosis (arterial wall stiffening) components of vascular aging (2) and detect individuals with early vascular aging (3). Aortic pulse wave velocity (aoPWV), a measure of aortic stiffness, has been found to be an independent predictor of cardiovascular events and all-cause mortality (4). Carotid-femoral pulse wave velocity (cfPWV) is the arteriosclerosis-related index calculated from hemodynamic data that is most widely used in outcome studies to date and is recommended by clinical guidelines for cardiovascular risk stratification (5, 6). However, skilled operators are required to acquire the data needed to calculate aoPWV and cfPWV, and, hence, their applicability is limited to specialized clinics. As a result, other hemodynamics-based indices have been proposed as alternative surrogates for arterial stiffness that can be calculated from data, which can be acquired in nonspecialized centers, and even in daily life using wearable devices (2, 7).

In recent years, the use of in silico data to study clinically relevant problems has been gaining momentum (8–12). In silico data avoid the expense, time, and challenge of collecting large in vivo datasets, simultaneously at several body sites, and measuring reference properties precisely (e.g., aortic stiffness). Previous studies have examined a few indices of vascular aging using in silico pulse wave (PW) data (13–19), but a comprehensive assessment of the most frequently used indices in clinical population studies using the same in silico PW dataset has not yet been conducted.

The aim of this study was to assess, in silico, the accuracy of all noninvasive indices of vascular aging that can be measured from pulse wave (PW) signals (20) and have known age-related changes, as identified in recent review articles on vascular aging (2, 7). The study used an in silico dataset of PWs simulated in 3,837 virtual healthy subjects aged 25–75 yr old (10), which provides precise theoretical values of arterial wall stiffness for each virtual subject. The tools used to create all the figures and tables in this study are freely available, allowing for the development and testing of other algorithms to assess vascular deterioration from PW signals.

## METHODS

### In Silico Data

**Figure 1.**
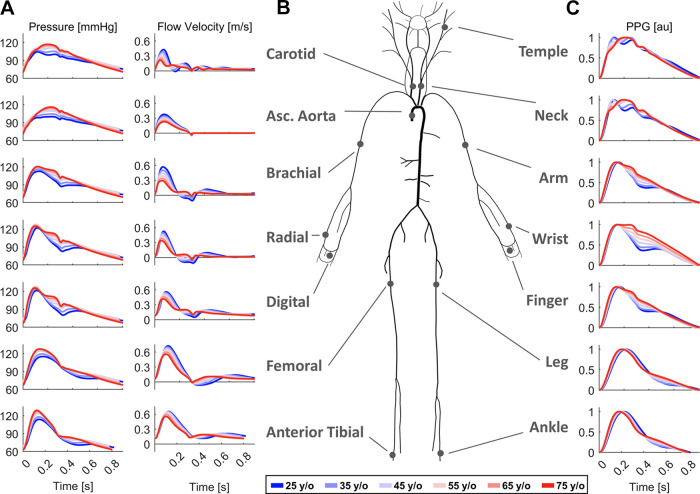
*A*: simulated pressure and flow velocity waveforms at the measurement arterial sites used in this study. *B*: schematic of the one-dimensional blood flow model that produced the in silico data. *C*: photoplethysmogram (PPG) waveforms at several body sites. Each waveform is shown for the baseline subject in each age decade from 25 to 75 yr (see Ref. 10 for details).

This study used a database of simulated blood pressure, blood flow velocity, luminal area, and photoplethysmogram (PPG) waveforms from the aorta and larger arteries of the head, neck, torso, and limbs in 3,837 virtual subjects distributed spanning six-age decades (25–75 yr old) (Fig. 1) (10). Normal aging was simulated by adjusting input cardiovascular parameters in a one-dimensional model of blood flow in 116 systemic arterial segments (Fig. 1*B*), in line with typical parameters for each age obtained from a comprehensive review of the clinical literature. The PW signals in this database exhibit age-related changes in morphology that are consistent with those observed in vivo, such as an increase in pulse pressure, a decrease in pulse pressure amplification, and the disappearance of the diastolic peak in the arm PPG waveforms (Fig. 1, *A* and *C*).

**Figure 2.**
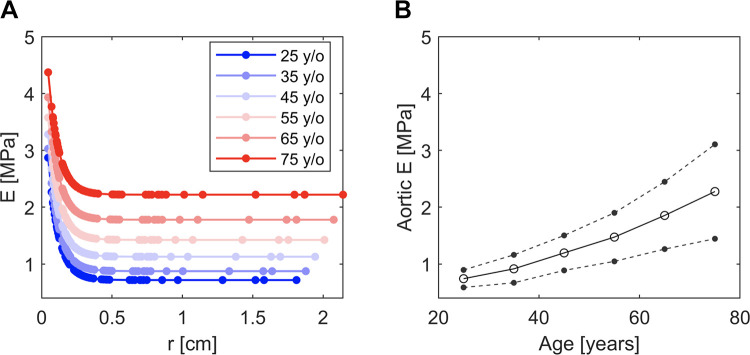
*A*: Young’s modulus (*E*) as a function of luminal arterial radius (*r*) for the baseline subject in each age decade. *B*: variation of the averaged *E* along the aorta (calculated using *Eq. 2*) with age for all virtual subjects. Solid line, mean values; dashed lines, ±1SD.

For every virtual subject, the database offers the precise theoretical value of Young’s modulus (*E*) of the wall of each arterial segment. This property is intrinsic to the vascular wall and represents its stiffness, which is often unavailable in in vivo PW datasets. *E* is defined as the ratio of arterial wall circumferential (or hoop) stress to arterial wall circumferential strain. It is an effective Young’s modulus that lumps the different constituent parts of the wall (21). In the in silico PW database, *E* is calculated using the empirical formula introduced by Olufsen (22),

(*1*) $$\frac{Eh}{r}=k_{1}\;\exp\left(k_{2}r\right)+k_{3},$$

where *h* is the wall thickness, *r* is the diastolic radius, and *k*_1_, *k*_2_, and *k*_3_ are empirical constants describing, respectively, the stiffness of smaller arteries, the point of transition in stiffness between smaller and lager arteries, and the stiffness of larger arteries. *k*_3_ was determined through an optimization process, aimed at calculating a specific value for each virtual subject by minimizing the absolute difference between desired and theoretical carotid-femoral PWV. *r*, *k*_1_, *k*_2_, and *k*_3_ are known input parameters for each subject in the database, so *Eh* is also an input parameter for each arterial segment. Figure 2*A* shows the relationship between normal *E* and *r* for each age decade in the dataset, with a constant thickness to radius (*h/r*) of 0.15 used in the calculation (23).

Aortic *E*, which is directly related to aortic compliance, plays a critical role in the arteriosclerosis component of vascular aging. The aorta accounts for more than half of the systemic circulation’s total compliance, i.e., its capacity to buffer pulsatile blood flow (24). The reduction in arterial compliance, and consequently this buffering capacity, is a major determinant of the increase in pulse pressure with hypertension (25). In this study, aortic Young’s modulus (*E*_Ao_) was defined as the sum of all aortic Young’s moduli (*E_j_*) weighted by the length of each aortic segment (*L_j_*),

(*2*) $$E_{\text{Ao}}=\sum_{i=1}^{10}\frac{L_{j}}{L_{\text{Ao}}}E_{j},$$

where *L*_Ao_ is the total length of the aorta, and *E_j_* was calculated for each of the 10 aortic segments in the model using *Eq. 1* [with *h/r* = 0.15 (23)], with the corresponding radius calculated as the average of the inlet and outlet radii of the segment. Figure 2*B* shows that *E*_Ao_ increases with age in our dataset of virtual subjects.

### Vascular Aging Indices

**Figure 3.**
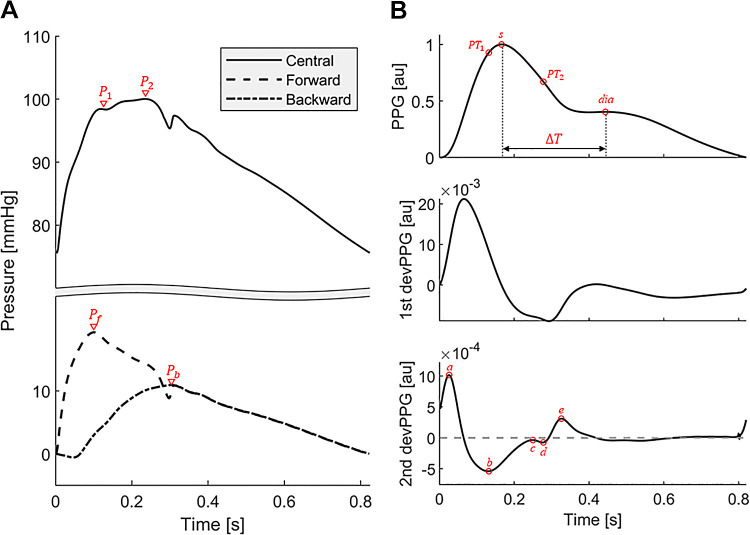
Pressure (*A*) and photoplethysmogram (PPG; *B*) fiducial points. P_1_, first inflection point/systolic peak; P_2_, 2nd inflection point/systolic peak, depending on the pressure wave type (45); P_f_, peak pressure of the forward-traveling pressure wave; P_b_, peak pressure of the backward-traveling pressure wave; s, PPG systolic peak amplitude; dia, PPG diastolic peak amplitude; PT_1_, PPG early systolic amplitude; PT_2_, PPG late-systolic amplitude; Δ*T*, time difference between systolic and diastolic PPG peaks; *a*, peak of early systolic positive wave of the second-derivative PPG; *b*, valley of early systolic negative wave of the second-derivative PPG; *c*, peak of late systolic reincreasing wave of the second-derivative PPG; *d*, valley of late systolic redecreasing wave of the second-derivative PPG; *e*, peak of early diastolic positive wave of the second-derivative PPG.

Table 1 describes the input data used to calculate each of the 19 indices evaluated in this study, common devices needed to acquire these data, and the mathematical formulas for calculating the indices. Figure 3 illustrates pressure and PPG fiducial points used in these calculations. We included all the indices from Climie et al. (2) that can be calculated using noninvasive PW signals, along with all PPG-derived indices listed in Charlton et al. (7) with strong evidence supporting their potential clinical utility. It is important to note that for the following indices, the input data used in this study differ from the data used in vivo: *1*) finger-toe pulse wave velocity (ftPWV) was calculated using the ankle PPG instead of toe PPG, which was unavailable in the in silico dataset; and *2*) central pulse pressure (cPP), augmentation pressure (AP), augmentation index (AIx), backward wave amplitude (Pb), and reflection magnitude (RM) were calculated directly from the aortic root pressure PW, instead of the central pressure PW obtained from the peripheral pressure PW using a transfer function. All six pulse wave velocity (PWV) indices were calculated using the foot-to-foot algorithm described by Gaddum et al. (46), with the foot defined as the intersection of the horizontal projection of the local minimum of the PW and the tangent projection of the local maximum gradient during systole. Arterial path lengths were measured directly from the arterial lengths of each virtual subject, which differs from the surface markings used in vivo for all PWV indices except for aoPWV. The PulseAnalyse algorithm (47) was used to extract fiducial points from a single pulse waveform to calculate the following related PW indices: cPP, brachial pulse pressure (bPP), AP, AIx, and PPG-based reflection index (RI_ppg_), stiffness index (SI_ppg_), aging index (AGI), augmentation index (AIx_ppg_), and *d*/*a*. Variations in all 19 in silico indices with chronological age were compared with corresponding variations in in vivo indices from the literature.

**Table 1.** Nineteen vascular aging indices studied

Index	Input Data	Common Device	Formula
Aortic pulse wave velocity (aoPWV) (26)	**Δ*x*_ao_**, distance between ascending and descending aorta; **Δ*t*_ao_**, time delay between the feet of the ascending and descending aorta flow PWs.	Magnetic resonance imaging	$\frac{\Delta x_{\text{ao}}}{\text{Δ}t_{\text{ao}}}$
Carotid-femoral pulse wave velocity (cfPWV) (27)	**Δ*x*_cf_**, distance between carotid and femoral arteries; **Δ*t*_cf_**, time delay between the feet of the carotid and femoral artery pressure PWs.	Applanation tonometer	$\frac{\Delta x_{\text{cf}}}{\Delta t_{\text{cf}}}$
Brachial-ankle pulse wave velocity (baPWV) (28, 29)	**Δ*x*_ba_**, distance between brachial and anterior tibial arteries; **Δ*t*_ba_**, time delay between the feet of the brachial and anterior tibial artery pressure PWs.	Cuff BP monitors and phonocardiogram	$\frac{\Delta x_{\text{ba}}}{\Delta t_{\text{ba}}}$
Carotid-brachial pulse wave velocity (cbPWV) (30)	**Δ*x*_cb_**, distance between carotid and brachial arteries; **Δ*t*_cb_**, time delay between the feet of the carotid and brachial artery pressure PWs.	Applanation tonometer	$\frac{\Delta x_{\text{cb}}}{\Delta t_{\text{cb}}}$
Carotid-radial pulse wave velocity (crPWV) (30)	**Δ*x*_cr_**, distance between carotid and radial arteries; **Δ*t*_cr_**, time delay between the feet of the carotid and radial artery pressure PWs.	Applanation tonometer	$\frac{\Delta x_{\text{cr}}}{\Delta t_{\text{cr}}}$
Finger-toe pulse wave velocity (ftPWV) (31)	**Δ*x*_ft_**, distance between digital and anterior tibial arteries; **Δ*t*_ft_**, time delay between the feet of the finger and ankle PPGs.	PPG sensor	$\frac{\Delta x_{\text{ft}}}{\Delta t_{\text{ft}}}$
Central pulse pressure (cPP) (32)	**SBP_c_**, aortic root systolic BP; **DBP_c_**, aortic root diastolic BP.	Applanation tonometer	SBP_c_ − DBP_c_
Brachial pulse pressure (bPP) (33)	**SBP_b_**, brachial systolic BP; **DBP_b_**, brachial diastolic BP.	Cuff BP monitor	SBP_b_ − DBP_b_
Augmentation pressure (AP) (34)	**P_1_**, early systolic BP peak at the aortic root; **P_2_**, reflected systolic BP peak at the aortic root (see Fig. 3*A*).	Applanation tonometer	P_2_ – P_1_
Augmentation index (AIx) (34)	**AP**, P_2_ − P_1_; **PP**, aortic root pulse pressure.	Applanation tonometer	$\frac{\text{AP}}{\text{PP}}$
Backward wave amplitude (P_b_) (35), (36)	**P_b_**, backward wave* (see Fig. 3*A*).	Doppler ultrasound and applanation tonometer	max (P_b_) – min (P_b_)
Reflection magnitude (RM) (35)	**P_b_**, backward wave*; **P_f_**, forward wave** (see Fig. 3*A*).	Doppler ultrasound and applanation tonometer	$\frac{\max\left({\text{P}}_{\text{b}}\right)-\text{min}({\text{P}}_{\text{b}})}{\max\left({\text{P}}_{\text{f}}\right)-\text{min}({\text{P}}_{\text{f}})}$
Cardio-ankle vascular index (CAVI) (37)	*c*_1_ and *c*_2_, empirical coefficients (38); **SBP_b_**, brachial systolic BP; **DBP_b_**: brachial diastolic BP; **bPP**, brachial pulse pressure; **haPWV**, PWV measured between ascending aorta and anterior tibial pressure PWs.	Cuff BP monitors and phonocardiogram	$c_{1}\;ln\left(\frac{{\text{SBP}}_{\text{b}}}{{\text{DBP}}_{\text{b}}}\right)\times \frac{2\rho }{\text{bPP}}\times {\text{haPWV}}^{2}+c_{2}$
Carotid distensibility coefficient (DC) (39)	**Δ*A***, change in carotid artery luminal area in a cardiac cycle; ***A*_dia_**, carotid artery diastolic luminal area; **bPP**, brachial pulse pressure.	Ultrasound-based echo tracking and cuff BP monitor	$(\frac{\Delta A}{A_{\text{dia}}})/\text{bPP}$
PPG-based reflection index (RI_ppg_) (40)	***s***, digital PPG systolic amplitude; ***dia***, digital PPG diastolic peak amplitude (see Fig. 3*B*).	PPG sensor	$\frac{s}{dia}$
PPG-based stiffness index (SI_ppg_) (41)	***H***, subject height; **Δ*T***, time difference between the systolic and diastolic peaks of digital PPG signal (see Fig. 3*B*).	PPG sensor	$\frac{H}{\Delta T}$
Aging index (AGI) (42)	***a***, peak of early systolic positive wave of the second-derivative digital PPG; ***b***, valley of early systolic negative wave of the second-derivative digital PPG; ***c***, peak of late systolic reincreasing wave of the second-derivative digital PPG; ***d***, valley of late systolic redecreasing wave of the second-derivative digital PPG; ***e***, peak of early diastolic positive wave of the second-derivative digital PPG (see Fig. 3*B*).	PPG sensor	$\frac{b-c-d-e}{a}$
PPG-based augmentation index (AIx_ppg_) (43)	**PT_1_**, digital PPG early systolic peak; **PT_2_**, digital PPG late systolic peak (see Fig. 3*B*).	PPG sensor	$\frac{{\text{PT}}_{2}}{{\text{PT}}_{1}}$
*d*/*a* (44)	***a***, peak of early systolic positive wave of the second-derivative digital PPG; ***d***, valley of late systolic redecreasing wave of the second-derivative digital PPG (see Fig. 3*B*).	PPG sensor	$\frac{d}{a}$

The following information is provided for each index (*1st column*): input data from each virtual subject used to calculate the index in this study (*2^nd^ column*), common device(s) needed to acquire these data in vivo (*3^rd^ column*), and mathematical formula for calculating the index from the in silico input data (*4^th^ column*). *First column* also shows references with further details for *columns 2* to *4*. BP, blood pressure; DBP, aortic root diastolic blood pressure; P, aortic root pressure PW; PPG, photoplethysmogram; PW, pulse wave; ρ, blood density; *U*, aortic flow velocity pulse wave. *${\text{P}}_{\text{b}}=\frac{1}{2}\sum \left[\left(\text{dP}-\rho cdU\right)\right]$; **${\text{P}}_{\text{f}}=\frac{1}{2}\sum \left[\left(\text{dP}+\rho cdU\right)\right]$. *c*, pulse wave velocity calculated using $c=\frac{1}{\rho }\sqrt{\frac{\sum {\text{dP}}^{2}}{\sum \text{d}U^{2}}}$.

### Statistical Analysis

Both Spearman’s (*r*_s_) and Pearson’s (*r*_p_) correlation coefficients were used to quantify the association between each PW index and *E*_Ao_ for each virtual subject. Pearson’s correlation measures the strength of a linear relationship between two variables, whereas Spearman’s correlation shows the monotonic relationship between two variables. A subgroup analysis was performed between young (25 yr old) and elderly (75 yr old) subjects to investigate any differences in correlations between these age groups.

Bland–Altman plots were obtained for all six PWV indices using the theoretical PWV calculated at the aortic root (aoPWT_t_) as reference,

(*3*) $$aoPWV_{t}=\sqrt{\frac{2Eh}{3\rho r}},$$

with *Eh* calculated using *Eq. 1* from the luminal radius, *r*, at the aortic root, and ρ = 1,060 kg/m^3^ the blood density used to run all virtual subject’s simulations. Bland–Altman plots illustrate the bias (i.e., mean error) and limits of agreement (i.e., range within which 95% of errors are expected to lie) between two measurement techniques.

### Sensitivity Analysis

The determinants of the indexes were assessed using the relative sensitivity index (I), which indicates the percentage of variation in a PW index resulting from independently changing a model input parameter by one standard deviation (SD) from its baseline value in the 25- to 75-yr-old baseline subjects (17),

(*4*) $$I=\frac{1}{n}\sum \left(\frac{V-\bar{V}}{\bar{V}}\frac{1}{v}\right)\times 100,$$

where *n* = 72 is number of tested virtual subjects, *V* is the value of each index, $\bar{V}$ is the baseline value of each index, and *v* represents variations (+1SD or −1SD) for each input parameter obtained from the clinical data reported by Charlton et al. (10). The input parameters studied were divided into two categories: cardiac and vascular properties. Cardiac properties included heart rate, stroke volume, and duration of systole. Vascular properties consisted of large arterial diameter, input PWV, and peripheral vascular resistance. The input PWV represents the theoretical cfPWV, which was used as an input parameter to optimize the desired *k_3_* value in *Eq. 1* for each virtual subject (10).

### Artificial Noise and Filtering

To investigate and compare the effect of real measurement noise on each index, white Gaussian noise was added to the in silico PW signals to simulate instrumental noise (48). Subsequently, noise reduction was performed to simulate real measurement conditions. All 19 indices were reextracted from the filtered noise-added PW signals.

**Figure 4.**
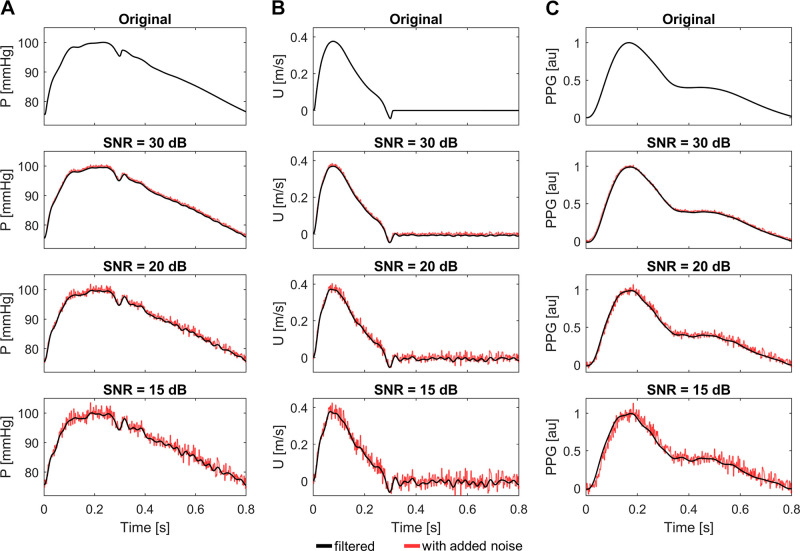
Effect of different levels of signal-to-noise ratios (SNRs) on the aortic root pressure waveform (P; *A*), aortic root flow velocity waveform (*U*; *B*), and digital photoplethysmogram waveform (PPG; *C*). Red lines, waveforms with noise; black lines, filtered waveforms.

Signals with various levels of interference were obtained by superimposing noises of different intensities on the original PWs. A bandpass filter was then used for signals with different noise, i.e., eliminating the content of frequencies >35 and <0.0665 Hz in the noise-added signal (47). The intensity of the noise was measured using the signal-to-noise ratio (SNR) formula,

(*5*) $$\mathrm{SNR=}\frac{PW_{\mathrm{signal}}}{PW_{\mathrm{noise}}},$$

where *PW*_signal_ and *PW*_noise_ are the average power of the signal and the noise present in the signal, respectively. Three levels of noise intensity were generated by setting SNR to 15, 20, and 30 dB, enabling the evaluation of the stability of each index under varying levels of noise interference. Figure 4 illustrates the impact of added noise and filter processing on the pressure, flow velocity, and PPG waveforms.

## RESULTS

### Evolution of Indices with Age

**Figure 5.**
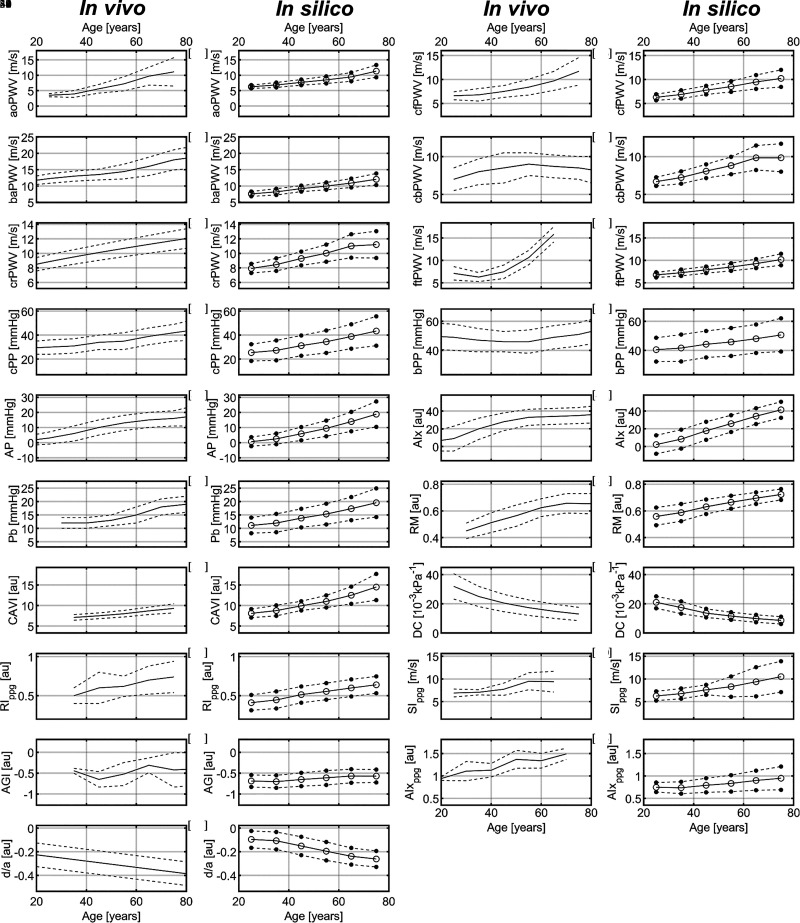
Comparison of chronological age variation in vascular aging indices calculated from simulated waveforms (in silico) and waveforms collected from real subjects (in vivo). Indices are described in Table 1. Solid lines, mean values; dashed lines, ±1SD. In vivo data were obtained from Refs. 27, 29–31, 33, 34, 38–41, 44, 49–52, as specified in the plots.

The vascular aging indices obtained from the in silico PW dataset exhibited similar variations with chronological age to those observed in corresponding indices calculated from in vivo PW datasets (Fig. 5). Simulated PW indices showed similar trends and, in most cases, similar absolute values. The exceptions were the ftPWV and bPP indices. ftPWV showed a slight decrease before the age of 40, followed by an exponential increase trend with age in vivo, differing from its linear growth trend in the in silico dataset. Similarly, bPP initially decreased and then increased with age in vivo, contrasting with its age-dependent increase throughout in the in silico dataset.

### Correlation of Indices with Aortic Stiffness

**Figure 6.**
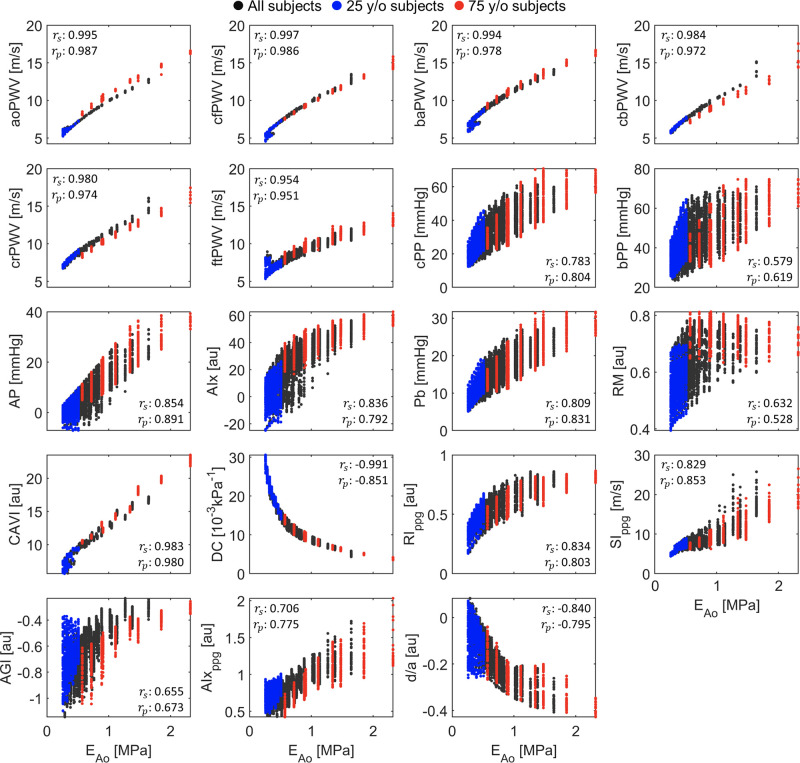
Relationship between each vascular aging index and aortic Young’s modulus (*E*_Ao_) for all virtual subjects (black), 25-yr-old subjects (blue), and 75-yr-old subjects (red). See Table 1 for a description of each index. Spearman’s correlation coefficients (*r*_s_) and Pearson’s correlation coefficients (*r*_p_) are provided for all subjects in each plot. See Table 2 for the *r*_s_ and *r*_p_ values for young and elderly subjects.

When examining the whole cohort, all six PWV indices and CAVI showed strong linear correlations with aortic Young’s modulus (*E*_Ao_) (*r*_p_ > 0.95) (Fig. 6). cfPWV displayed the strongest nonlinear correlation with *E*_Ao_ (*r*_s_ = 0.997) among all indices, as shown in Table 2. Carotid DC exhibited a strong negative nonlinear correlation with *E*_Ao_ (*r*_s_ = −0.991). Among all the indices, bPP and RM had the weakest correlation with *E*_Ao_ (*r*_s_ and *r*_p_ < 0.63). In addition, Table 2 demonstrated correlation results between each index and *E*_Ao_ in young and elderly subjects. Elderly subjects exhibited greater variability in the values of all indices, except for RM and carotid DC, than young subjects (Fig. 6). Overall, *r*_s_ and *r*_p_ absolute values in elderly subjects were similar to the corresponding values for the whole cohort and larger than the corresponding values for young subjects (Table 2). In young subjects, the first five PWV indices and CAVI still demonstrated strong linear correlations with *E*_Ao_ (*r*_p_ > 0.85), though smaller than those observed for elderly subjects and all subjects combined (*r*_p_ > 0.97). The exception was ftPWV, for which *r*_p_ dropped to 0.57. Carotid DC displayed a stronger negative nonlinear correlation with *E*_Ao_ when calculated in young subjects rather than in elderly subjects. Among the PPG-based indices, SI_ppg_ showed the strongest linear correlation with *E*_Ao_ among the three cohorts (*r*_p_ > 0.85) and *d*/*a* showed the highest nonlinear correlation with *E*_Ao_ for all subjects combined (*r*_s_ = −0.84). Lastly, AP, AIx, RM, AGI, and *d*/*a* demonstrated the weakest associations with *E*_Ao_ in young subjects $\left(|r_{\text{s}}\right|$ and $\left|r_{\text{p}}|<0.32\right)$ among all indices.

**Table 2.** Spearman’s and Pearson’s correlation coefficients between each of the 19 vascular aging indices and aortic Young’s modulus for all subjects

	Spearman’s Correlation Coefficient			Pearson’s Correlation Coefficient		
	All subjects	25 y/o subjects	75 y/o subjects	All subjects	25 y/o subjects	75 y/o subjects
cfPWV	0.997	0.964	0.993	0.986	0.955	0.994
aoPWV	0.995	0.936	0.976	0.987	0.970	0.991
baPWV	0.994	0.917	0.992	0.978	0.902	0.988
DC	−0.991	−0.981	−0.962	−0.851	−0.954	−0.932
cbPWV	0.984	0.957	0.964	0.972	0.983	0.985
CAVI	0.983	0.882	0.962	0.980	0.847	0.981
crPWV	0.980	0.922	0.957	0.974	0.956	0.990
ftPWV	0.954	0.600	0.967	0.951	0.567	0.947
AP	0.854	0.254	0.873	0.891	0.213	0.873
*d*/*a*	−0.840	−0.162	−0.675	−0.795	−0.206	−0.699
AIx	0.836	0.276	0.859	0.792	0.220	0.799
RI_ppg_	0.834	0.649	0.840	0.803	0.643	0.790
SI_ppg_	0.829	0.899	0.871	0.853	0.851	0.886
P_b_	0.809	0.622	0.783	0.831	0.646	0.802
cPP	0.783	0.600	0.764	0.804	0.639	0.781
AIx_ppg_	0.706	0.375	0.875	0.775	0.446	0.807
AGI	0.655	0.223	0.900	0.673	0.318	0.832
RM	0.632	0.309	−0.130	0.528	0.258	−0.198
bPP	0.579	0.505	0.651	0.619	0.551	0.695

Participants comprise 25- and 75-yr-old (y/o) subjects. Data are sorted in descending order based on Spearman’s correlation coefficients of each index for all subjects. See Table 1 for definitions.

**Figure 7.**
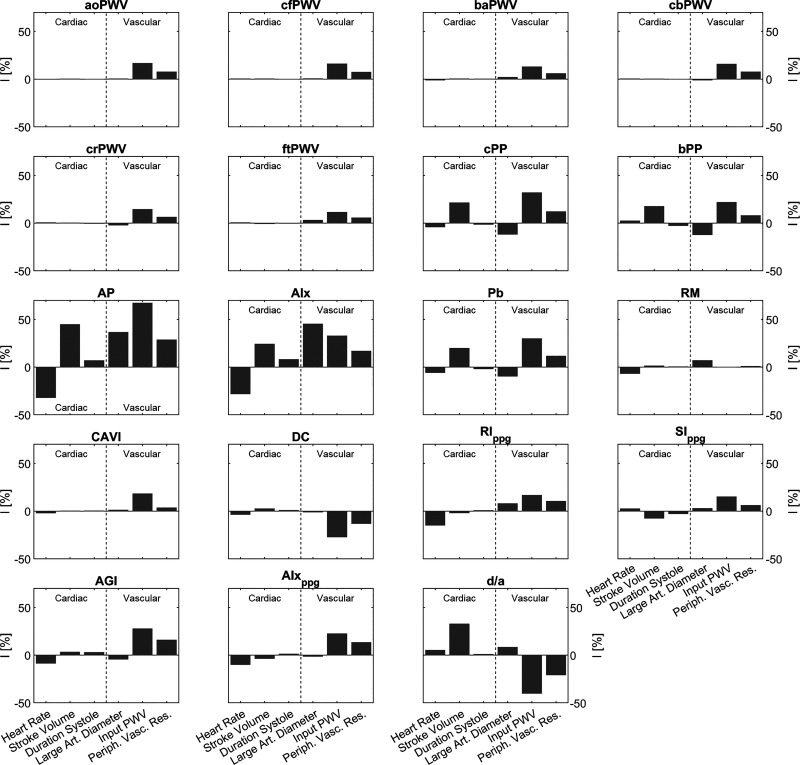
Physiological determinants of all indices assessed using the relative sensitivity index (I) calculated for three cardiac properties and three vascular properties.

Changes in input PWV resulted in variations in the values of each vascular aging index studied, by at least 11.4%, as measured by the relative sensitivity index (I) (Fig. 7), except for RM (|I*|* = 0.1%). Input PWV was the main determinant of all indices except for AIx and RM. The index that was mostly affected by variations in input PWV was AP (I = 67.0%), but this index was also considerably affected by other cardiovascular properties. Indeed, other cardiovascular properties, in addition to input PWV, affected most of the indices studied, resulting in greater absolute I values than input PWV for AIx and RM. Heart rate was found to be a main determinant of AP and AIx (|I*|* > 28.0%) and the second determinant of RI_ppg_ (|I*|* = 14.7%); stroke volume played a key role in cPP, bPP, AP, AIx, P_b_, and *d*/*a* (I > 17.5%); and duration of systole considerably affected AP and AIx (I > 6.8%). Large artery diameter was the first determinant for AIx and RM (I = 45.1 and 6.9%, respectively) and the third determinant for AP (I = 36.4%). Lastly, peripheral arterial resistance was the second determinant of all PWV indices, DC, AGI, and AIx_ppg_ (|I| > 12.9%), after input PWV, and was the third determinant of *d*/*a* (|I| = 20.7%).

**Figure 8.**
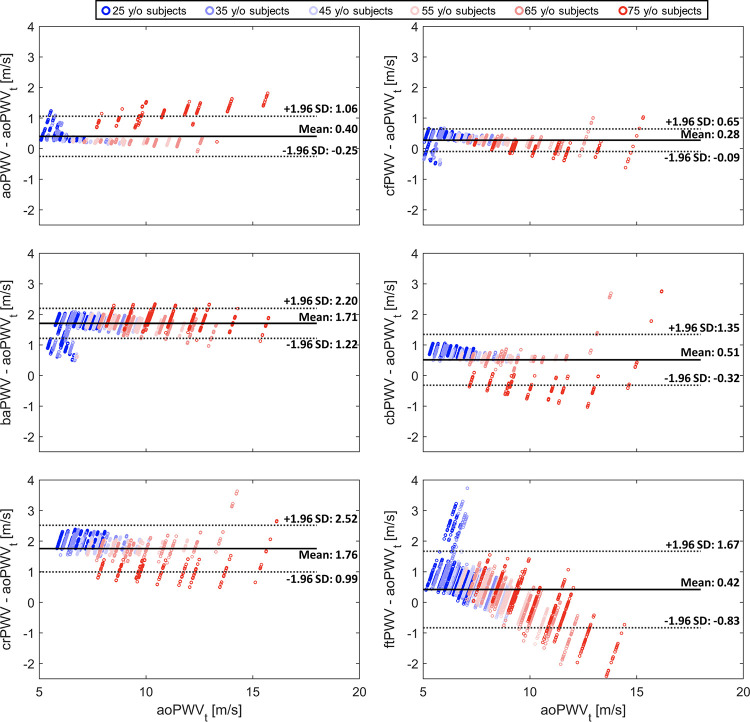
Bland–Altman plots comparing the six pulse wave velocity indices with the theoretical aortic pulse wave velocity (aoPWVt) calculated using *Eq. 3*. Each circle represents data from a virtual subject, and color code indicates age of each subject. Solid lines, mean difference values; dashed lines, ±1.96 SD.

Bland–Altman analysis was conducted to investigate the agreement between the six measured PWV indices and aoPWV_t_ calculated using *Eq. 3*. All measured PWV indices overestimated aoPWV_t_ on average (Fig. 8). The mean differences between measured aoPWV and aoPWV_t_ and between measured cfPWV and aoPWV_t_ approached zero (mean = 0.4 and 0.3 m/s, respectively), with relatively narrow bands of limits of agreement (1.96 SD = 0.66 and 0.37 m/s, respectively). This indicates that both aoPWV and cfPWV can accurately estimate aoPWV_t_. Measured baPWV and crPWV also showed relatively narrow bands of limits of agreement (1.96 SD = 0.5 and 0.8 m/s, respectively) but with a much larger mean difference (1.7 and 1.8 m/s, respectively), indicating that baPWV can precisely estimate aoPWV_t_. cbPWV and ftPWV displayed relatively larger bands of limits of agreement (1.96 SD > 0.8 m/s), although the mean values were smaller than baPWV and crPWV (<0.5 m/s), showing small precision but reasonable accuracy, when examining the whole cohort. All PWV indices displayed higher agreement with aoPWV_t_ for young subjects, as indicated by more points falling within the limits of agreement when aoPWV_t_ was small.

### Effect of Noise

**Figure 9.**
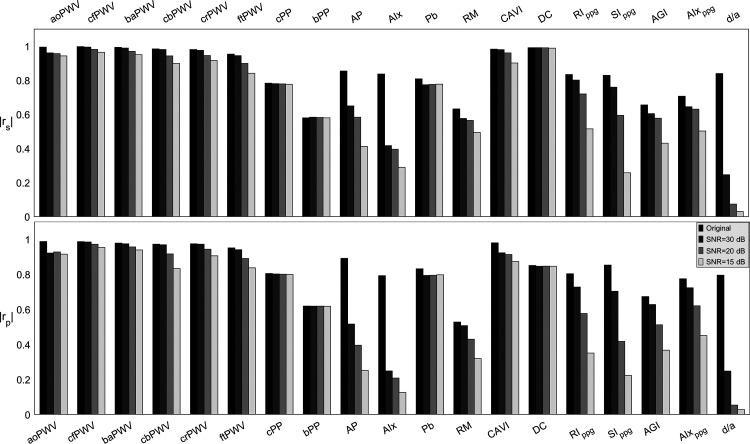
Variation in the absolute value of Spearman’s (*r*_s_) and Pearson’s (*r*_p_) correlation coefficients between each vascular aging index and aortic Young’s modulus (*E*_Ao_) for different signal-to-noise ratios (SNRs).

Decreasing levels of SNR resulted in increasing fluctuations in the filtered PW morphologies for all virtual subjects (Fig. 4), which reduced the Spearman’s (*r*_s_) and Pearson’s (*r*_p_) correlation coefficients between each index and *E*_Ao_ (Fig. 9). The reflection pressure indices (i.e., AP, AIx, and RM) and the PPG-based indices (RI_ppg_, SI_ppg_, AGI, AIx_ppg_, and *d*/*a*) were highly susceptible to noise disturbances, with AP, AIx, and *d*/*a* being susceptible even at low levels of noise (SNR = 30 dB). Indices such as cPP, bPP, P_b_, and carotid DC were relatively robust to noise interference; in these indices, *r*_s_ and *r*_p_ decreased by just under 0.03 for the lowest (15 dB) SNR studied. All PWV indices and CAVI were only moderately affected by SNR ≥ 20 dB (*r*_s_ and *r*_p_ reduced to 0.90 at most), but relatively significantly affected when SNRs dropped to 15 dB (*r*_s_ and *r*_p_ reduced to 0.84 at most).

## DISCUSSION

We have assessed the ability of 19 indices of vascular aging to estimate changes in aortic wall stiffness on nearly 4,000 virtual healthy subjects aged 25–75 yr. All the indices examined can be calculated from noninvasive PW data, are known to change with age, and include PPG-based indices that can be measured in daily life using wearable devices (2, 7). This is the first study to compare all 19 indices using a single dataset that included exact values of the aortic Young’s modulus and other hemodynamic measures. This allowed us to assess the correlation of each index with a direct measure of aortic stiffness and investigate how confounding cardiac and vascular properties might affect each index. Furthermore, all the hemodynamic data used in the study were free from measurement errors, enabling us to analyze how each index was impacted by different levels of measurement errors added in a fully controlled manner. Our results have shown that PWV indices, cardio-ankle vascular index (CAVI), and the carotid distensibility coefficient (DC) are all highly reliable indicators of the vascular stiffening process that occurs with aging, with CAVI and DC being more robust to noise interference than PWV indices. Among the indices that can be calculated in nonspecialized centers, CAVI (calculated from a cuff blood pressure monitor) and ftPWV, RI_ppg_, SI_ppg_, AGI, AIx_ppg_, and *d*/*a* (calculated from PPG signals) demonstrated strong associations with aortic stiffness that were robust to noise disturbances for CAVI and ftPWV.

Our study included cfPWV, the PW index recommended by clinical guidelines for cardiovascular risk stratification (5, 6), and aoPWV, which is measured directly in the aorta (26). Both indices were found to accurately estimate the theoretical aortic PWV, albeit with a slight overestimation, and consequently demonstrated strong correlations with aortic Young’s modulus, with little confounding from cardiac properties, arterial diameter, or peripheral vascular resistance. The slightly weaker correlation of aoPWV with aortic stiffness, compared with cfPWV, may be attributed to the following reasons. First, aoPWV measures the velocity of PW propagation over a short portion of the aorta, i.e., from the ascending to the descending aorta. In contrast, cfPWV accounts for the much longer portion of the aorta that connects the carotid and femoral sites where the PW is measured to calculate cfPWV. As a result, cfPWV contains information about aortic stiffness for the entire aorta, similar to the weighted aortic Young’s modulus *E*_Ao_ used as the reference for aortic stiffness in our study. Second, the shorter distance between the two recording points in the ascending aorta used to calculate aoPWV, compared with the distance between the carotid and femoral sites used for cfPWV, results in considerably smaller PW transit times involved in the calculation of aoPWV. Consequently, the same absolute measurement error in the calculation of the aoPWV and cfPWV transit times will lead to larger relative errors in transit time and, hence, PWV, for the aoPWV index compared with cfPWV.

We found that other PWV indices calculated from peripheral PW data (baPWV, cbPWV, crPWV, and ftPWV) were also able to capture aortic stiffness with either reasonable precision or accuracy, and without confounding influences. This is consistent with previous in vivo studies showing strong correlations between this peripheral PWV indices and cfPWV or aoPWV (28, 53–56).

The blood pressure-related indices studied here (i.e., cPP, bPP, AP, AIx, P_b_, and RM) have been proposed as indicators of arterial stiffness in previous studies, because of their ability to be calculated from measurements acquired in a single arterial site, unlike PWV indices. However, these indices showed the weakest correlations with aortic Young’s modulus, as they were affected by cardiac and other vascular properties, in addition to arterial stiffness. Consistent with our in silico-based findings, previous in vivo studies have also reported confounding influences of heart rate on AIx (57), stroke volume on arterial PP (58), and stroke volume on P_b_ (59). Notably, cPP, bPP, and P_b_ remained stable under noise interference, suggesting their potential for practical applications.

In addition to ftPWV, other PPG-based PW indices (i.e., RI_ppg_, SI_ppg_, AGI, AIx_ppg_, and *d*/*a*) demonstrated potential for assessing aortic stiffness using a single PPG signal obtained at a peripheral site. However, these indices were found to be confounded by cardiac and vascular properties, and highly susceptible to noise disturbances. These limitations should be considered when designing a PPG-based wearable device for vascular aging assessment, since the PPG signal is susceptible to various issues that can compromise signal quality (60–62).

Unlike other studied indices, the correlation coefficients of AP, AIx, RI_ppg_, SI_ppg_, AGI, AIx_ppg_, and *d*/*a* with aortic Young’s modulus showed considerable decreases under excessive noise perturbations (15 dB), even with the filtering of the input PW signals used for their calculations. These seven indices relied on fiducial points derived from a single PW signal using its first and second derivatives, which made them more susceptible to errors. Since an excess of noise can induce considerable fluctuations in the original PW signal, even after filtering procedures, these indices were particularly affected. Conversely, other indices only require the identification of PW maximum and/or minimum points, which are less affected by minor noise interference.

Some of the discrepancies observed between in vivo and in silico indices in Fig. 5 are a direct consequence of the following facts. The ftPWV discrepancy is the result of the nonlinear fitting used by Hallab et al. (31) to describe the variation of ftPWV with age from 12 to 95 yr. A linear fitting from 25 to 75 yr (the in silico age group) would result in a similar trend to the one obtained in silico, since all 75-yr-old in vivo subjects had a ftPWV < 14 m/s. Furthermore, the in silico ftPWV was calculated using the ankle PPG signal, rather than the toe signal used in vivo (since the in silico toe PPG was unavailable). The bPP discrepancy can be attributed to the brachial systolic BP generated by our simulated model being lower than the values reported in the literature at 25 and 35 yr old (34). Furthermore, the in vivo datasets of aoPWV, crPWV, ftPWV, and RI_ppg_ included subjects with cardiovascular diseases, whereas the virtual subjects were all generated under healthy conditions. In addition, the in vivo datasets for aoPWV, cfPWV, ftPWV, RI_ppg_, AGI, and AIx_ppg_ did not differentiate subjects by biological sex (27, 40, 49, 50), while only male subjects were simulated in the in silico dataset. Furthermore, the in vivo dataset for *d*/*a* included exclusively female subjects (44).

An obvious limitation of our study is that we used in silico data obtained from virtual subjects. This choice was motivated by the lack of a large dataset of in vivo pressure, flow, area, and PPG PW signals acquired simultaneously at all the body sites required to compute the 19 indices studied here. In addition, it is challenging to measure in vivo reference values for aortic stiffness in each subject. Despite relying on modeling hypothesis, the in silico PW signals produced stiffness indices that displayed similar age-related changes to those reported in in vivo studies. This provides confidence that our selected dataset generates hemodynamic data with realistic age-related changes. It is worth noting that the dataset used in our study represents a sample of healthy adults; therefore, the findings and conclusions drawn from our study might not be valid when assessing cohorts with underlying pathophysiological conditions. Another limitation arises with the use of *Eq. 1*, which neglects arterial characteristics influencing wall elasticity, potentially resulting in modeling inaccuracies (63). Nevertheless, the aortic Young’s modulus in virtual subjects of different age groups exhibits a notable trait from in vivo datasets: subjects of different age groups can have similar values. For instance, among the virtual subjects, there are 75-yr-old subjects with a Young’s modulus akin to that of 45-yr-old virtual subjects (see Fig. 2*B*). Furthermore, we only studied the effect of instrumental noise and did not consider the impact of physiological noise (e.g., motion artifacts), which is commonly encountered in the measurement of physiological signals (64, 65). Lastly, only five PPG-based indices were studied because of their potential clinical utility as highlighted in a recent review article (7). However, many indices based on PPG signals or wearable devices have not been sufficiently studied, which constitutes an area of future research exploration.

### Perspectives

Our in silico study could provide valuable insights for designing future clinical studies to noninvasively assess vascular aging. We found that most of the indices studied here were determined in part by cardiac properties and other vascular properties, in addition to aortic stiffness. These findings indicate the need for future studies to evaluate the performance of PW indices during changes in cardiac and vascular properties. Furthermore, we observed that the performance of all indices, except for RM, DC, and SI_ppg_, considerably decreased when analyzing only young subjects, as opposed to when analyzing the entire cohort of subjects aged 25–75 yr. For instance, the *r*_p_ value for ftPWV decreased from 0.95 when assessing the entire cohort to 0.57 when analyzing only the 25-yr-old subjects. Therefore, our study highlights the importance of assessing indices across a narrow age range to determine their usefulness in stratifying vascular aging.

This article is accompanied by the MATLAB code used to generate all figures and tables in the study, which should facilitate the development and testing of other PW indices and the algorithms used to calculate them (66).

### Conclusions

This study examined, for the first time, the ability of 19 indices related to vascular aging stiffness to assess aortic stiffness in healthy subjects. Overall, carotid-femoral PWV, CAVI, and carotid distensibility emerged as the most robust PW indices, showing a high correlation with aortic stiffness and stability against noise disturbances, making them reliable clinical indicators. Indices derived from blood pressure wave morphology showed weaker correlations with aortic Young’s modulus due to influence from other cardiovascular properties, although some exhibited resistance to noise interference. PPG-based indices measured at peripheral sites hold potential for assessing vascular aging in daily life with minimized noise disturbances during signal acquisition.

## DATA AVAILABILITY

All in silico data used in this study can be downloaded from: https://zenodo.org/records/3275625.

## SUPPLEMENTAL DATA

10.5281/zenodo.8070863 MATLAB code used to generate all figures and tables in the study can be downloaded from https://doi.org/10.5281/zenodo.8070863.

## GRANTS

This work was supported by British Heart Foundation Grants PG/15/104/31913 and FS/20/20/34626, Engineering and Physical Sciences Research Council Doctoral Training Partnership Grant EP/T517963/1, and Department of Health through the National Institute for Health Research Cardiovascular MedTech Co-operative at Guy’s and St Thomas’ National Health Service Foundation Trust Grant MIC-2016-019.

## DISCLOSURES

No conflicts of interest, financial or otherwise, are declared by the authors.

## AUTHOR CONTRIBUTIONS

J.A. conceived and designed research; J.H. performed experiments; J.H. analyzed data; J.H. and J.A. interpreted results of experiments; J.H. prepared figures; J.H. and J.A. drafted manuscript; J.H., M.N., P.H.C., and J.A. edited and revised manuscript; J.H., M.N., P.H.C., and J.A. approved final version of manuscript.

## ENDNOTE

At the request of the authors, readers are herein alerted to the fact that additional materials related to this manuscript may be found at https://zenodo.org/record/8070863. These materials are not a part of this article and have not undergone peer review by the American Physiological Society (APS). APS and the journal editors take no responsibility for these materials, for the website address, or for any links to or from it.
